# The impact of an exercise program on quality of life in older breast cancer survivors undergoing aromatase inhibitor therapy: a randomized controlled trial

**DOI:** 10.1186/s12955-019-1090-4

**Published:** 2019-01-18

**Authors:** Thais R. S. Paulo, Fabricio E. Rossi, Juliana Viezel, Giuliano T. Tosello, Sylvia C. Seidinger, Regina R. Simões, Ruffo de Freitas, Ismael F. Freitas

**Affiliations:** 1 State University of Sao Paulo, UNESP, School of Technology and Sciences, Rua Roberto Simonsen, 305, Presidente Prudente, Sao Paulo CEP 19060-900 Brazil; 2 Federal University of Rio Grande do Norte, UFRN, Natal, Brazil; 3 Federal University of Piauí, UFPI, Teresina, Brazil; 4 Federal University of São Paulo, USP, Sao Paulo, Brazil; 5 Federal University of Triângulo Mineiro, UFTM, Uberaba, Brazil; 6 Federal University of Goias, UFG, Goiânia, Brazil

**Keywords:** Public health, Combined exercise, Breast cancer, Menopause, Hormone therapy, Physical activity

## Abstract

**Background:**

This study evaluated the impact of an exercise program on quality of life in older breast cancer survivors undergoing aromatase inhibitor therapy.

**Methods:**

Older breast cancer survivors were randomized into two groups: combined training: resistance + aerobic exercise program for nine months (*n* = 18) or control group (*n* = 18). Quality of life was assessed by the questionnaires SF36, EORTC QLQ-C30, and EORTC QLQ-BR23 at baseline, and at three, six, and nine months. The exercise group performed 40 min of resistance exercises on machines followed by 30 min of aerobic training on a treadmill 3x/wk. Repeated measures ANOVA was used to compare the groups over time.

**Results:**

Significant time x group interactions and moderate to high effect sizes were found for physical functioning, physical health, bodily pain, general health perception, vitality, social functioning, fatigue, sleep disturbance, body image, and upset by hair loss, favoring the exercise group.

**Conclusion:**

This study demonstrated the potential benefits and high clinical relevance of exercise programs to improve quality of life in older breast cancer survivors undergoing aromatase inhibitor therapy.

## Introduction

Cancer is considered a public health problem in the majority of countries in the world [18]. Breast cancer is the most frequent cancer in women and the Globocan project estimated that 1.6 million new cases of breast cancer were diagnosed in 2012 worldwide [20]. In parallel, breast cancer mortality has declined in recent years due to early detection and improvement in treatment [18]. Approximately two and a half million breast cancer survivors live in the United States [39]. In Brazil, breast cancer mortality is considered to have stabilized in regions with a high socioeconomic level, however in regions with low socioeconomic levels the mortality rate has increased due to the absence of periodic exams and detection of the disease at an advanced stage, making the effectiveness of treatment more difficult [26].

Several factors have contributed to the improvement in breast cancer survival rate, among them the use of endocrine therapy, which could be responsible for reductions in the risk of local recurrence, distant metastasis, and death [17]. The first description of endocrine therapy was an oophorectomy for advanced breast cancer performed in 1895 by Dr. Beatson [5]. Approximately 80 years later, tamoxifen was the first effective endocrine drug to treat breast cancer with fewer side effects [11]. In the mid-1990s, the third generation of aromatase inhibitor therapy (AI) was developed, including Anastrozol, Letrozol, and Exemastano, which reduce circulating estrogen levels, depriving the tumor of the stimulus for growth [19]. AI are useful in menopausal women and those with estrogen receptor positive (ER+) tumors [19, 25] and are considered the gold standard treatment in postmenopausal women with breast cancer [10]. However, the use of AI may lead to several symptoms during and after therapy, consequently jeopardizing many aspects of quality of life (QoL) (e.g., changes in body composition – increased fat mass and decreased lean mass, osteoporosis, depression, anxiety, low self-esteem, fatigue, pain, and reduced physical fitness) [3, 33].

Hojan et al. [30] showed that exercise programs may improve QoL and reduce the adverse effects of endocrine therapy in premenopausal breast cancer patients. Cancer patients who performed aerobic exercise during and after treatment with radiotherapy and chemotherapy demonstrated measurable improvement in QoL, oxygen consumption, and body composition [27]. Findings from Leach and colleagues study (2016) suggest that longer duration (i.e., greater than 12 weeks) exercise programs during treatment and/or continuation of programs following treatment in breast cancer survivors may be indicated in order to facilitate improvement beyond preventing declines or maintenance of QoL, fitness, and fatigue.

Exercise programs can contribute to improving the outcomes of cancer medical treatment, and, in addition, are advisable for the prevention and treatment of many disorders [21, 35]. Aerobic and resistance exercise, either separately or in combination, have been shown to improve physical functioning and manage some symptoms in breast cancer patients [34, 54]. However, it is unclear whether older breast cancer survivors undergoing AI therapy can also benefit from physical activity. Therefore, the objective of the present study was to evaluate the impact of an exercise program on quality of life in older breast cancer survivors undergoing aromatase inhibitor therapy.

## Methods

### Study design and subjects

This randomized controlled clinical trial (RBR-9CBP8S) was performed according to the guidelines of the Declaration of Helsinki and the project was approved by the Ethics Research Group of the University (Protocol number: 6727715.1.0000.5402 / 2015).

According to the Medical records in the Oncology Department, a total of 348 registered breast cancer survivors were identified, including all types of treatment for cancer. For the present study, only postmenopausal breast cancer survivors undergoing aromatase inhibitor therapy were invited to participate, totaling 124 women.

The eligibility criteria included postmenopausal women aged between 50 and 80 years, using AI, with a diagnosis of stage I to III breast cancer, without musculoskeletal injuries, with physician clearance to participate in physical training, living in the city, returning a signed consent form to participate in the research, and not having participated in supervised physical exercise for at least 6 months prior to enrollment in the study.

For the present study, a power analysis was performed, based on the observation from a previous study that found improvement of 13.4 points in the overall quality of life assessed by the functional assessment of cancer therapy-breast (FACT-B) scale after 10 weeks of exercise training in postmenopausal breast cancer survivors [38]. Using a power (1-type II error) of 0.90 and a type I error of 0.05, according to PS software (see 3.1.2, Dupont and Plummer, http://biostat.mc.vanderbilt.edu/wiki/Main/PowerSampleSize), it was estimated that 11 subjects per group were needed. Considering a dropout rate, we over-recruited the number of the target sample to 18 women per group.

**Fig. 1:**
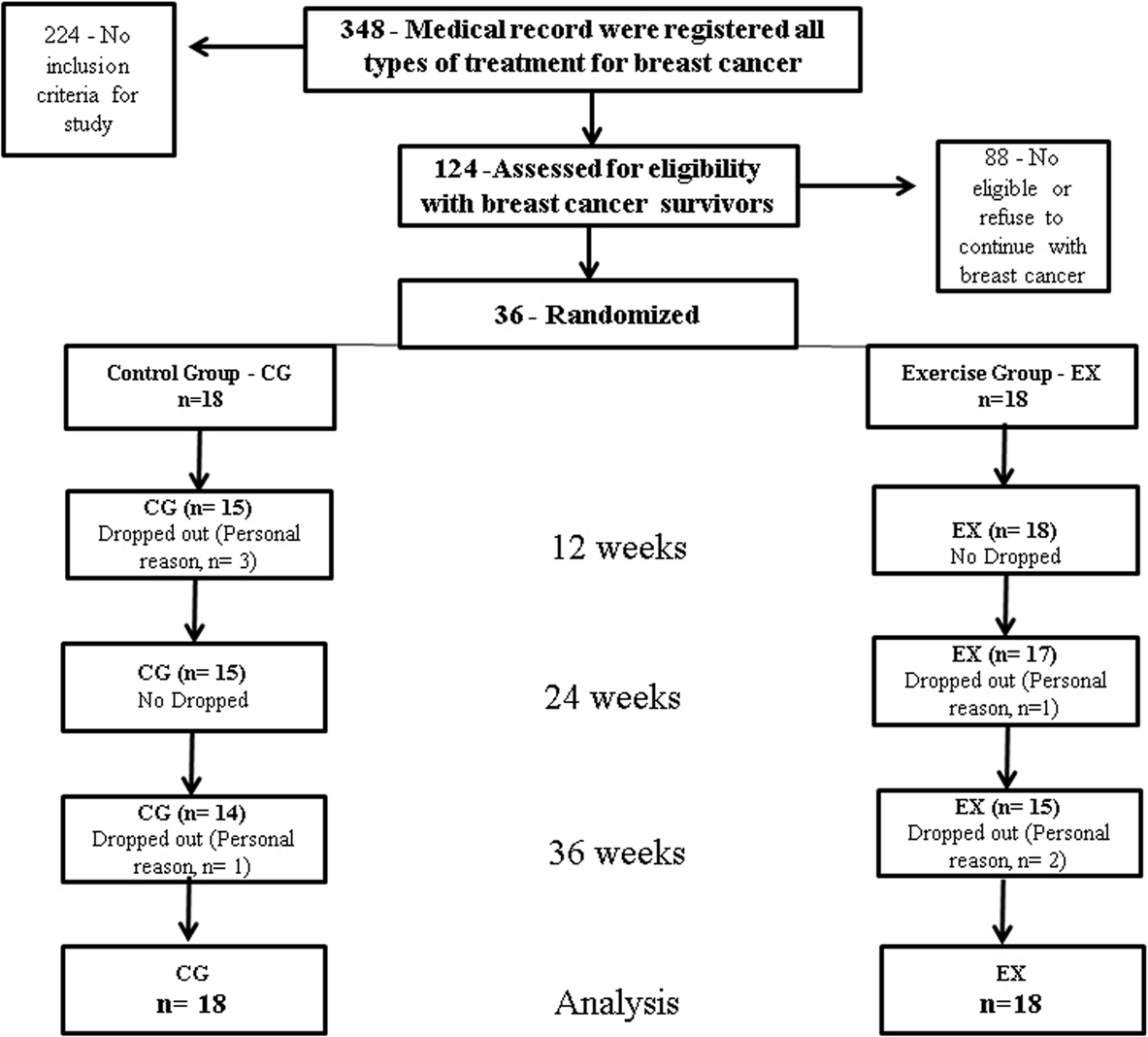
The trial profile of this study

A total of 36 women were included in the randomized clinical trial, 18 allocated to the control group (stretching) of breast cancer survivors (CG) and 18 allocated to the supervised combined exercise training (EX), demonstrated in Fig. 1.

### Physical training program of exercise group

The intervention lasted 9 months, with a frequency of three times a week, on nonconsecutive days, and all sessions were supervised by physical education professionals. The first 2 weeks were allocated as a familiarization period to the training protocol.

The combined training program consisted of resistance training followed by aerobic training. The resistance exercises were performed on a machine, lasting 40 min, with the following exercises: seated cable row; bench press; leg extension; leg press; leg curl; bridge; and plank. The intensity of resistance training was controlled by maximum repetition training zones, where the series were performed until momentary exhaustion of the patient [47].

Next the resistance exercise aerobic training was performed on a treadmill (Movement, LX-160, Fitness Equipment, Pompeia, São Paulo, Brazil), lasting 30 min, prescribed from the maximum heart rate (HR) within the target training zone, controlled by heart monitors (model S810i; Polar Electro, Kempele, Finland) during all exercise sessions. The aerobic protocol consisted of four progressive stages: [Stage 1: 1st to 8th weeks, 30 min/day, 60–65% HR max]; [Stage 2: 9th to 20th weeks, 30 min/day, 65–70% HR max]; [Stage 3: 21st to 30th weeks, 30 min/day, 70–75% HR max]; [Stage 4: 31st to 36th weeks, 30 min/day, 75–80% HR max]. After training, as a cool down, stretching exercises were performed to complete the protocol.

Participants in the exercise group were invited to attend health education lectures once per month, with 90 min duration per session. The topics discussed were related to breast cancer, health promotion, quality of life, physical activity, well-being, and mental health. These health education lectures were conducted by doctors, nutritionists, physical educators, and a psychologist.

### Control group

The control group was invited to participate in stretching and relaxation exercises, 2 times per week, with 45 min session durations, for 9 months, and the exercises were active, lasting 10–15 s each. The frequency of this class was not controlled and all participants could participate as and when they wanted.

### Anthropometric and quality of life measurements

To characterize the sample all participants answered a questionnaire including socio-demographic and clinical information. The assessment of body mass was carried out using a Filizola mechanical scale with a precision of 0.1 kg and a maximum capacity of 180 kg; height was measured using a fixed stadiometer of the Sanny brand with a precision of 0.1 cm and maximum length of two meters, according to the methodology proposed by Freitas Jr [22].

Quality of life (QoL) was measured using the European Organization for Research and Treatment of Cancer questionnaire entitled “Quality of life Questionnaire version 3.0” (EORTC QLQ-C30) and the European Organization for Research and Treatment of breast cancer module (EORTC QLQ-BR23). The EORTC QLQ-C30 is a 30-item scale that measures quality of life in cancer patients, who receive scores for global health status (2 items: health and global quality of life). The EORTC QLQ-C30 contains a symptom scale with pain, fatigue, nausea and vomiting, dyspnea, sleep disturbance, constipation, diarrhea, appetite loss, and financial difficulties scores (13 items: nausea and vomiting, pain, dyspnea, sleep, disturbance, appetite loss, constipation, diarrhea, and fatigue), and a functional scale with social functioning, physical functioning, cognitive functioning, and emotional functioning scores (15 items: strenuous activities, self-care, long/short walk, limitations at work, limitations in leisure, depression, worry, tension, and irritability). Each item is rated on a scale from 0 (not at all) to 4 (very much), with the exception of two items on the global health/QoL scale which use modified 7-point linear analog scales. According to the scores received, high scores on the symptom scale represent a high level of problems and high scores on the global health and functional scales indicate good quality of life [1].

The EORTC QLQ-BR23^22^ consists of 23 items, and is a tumor specific tool, which incorporates four functional scales - body image, sexual functioning, sexual enjoyment, and future perspective - and four symptom-oriented scales - systemic therapy side effects, breast symptoms, arm symptoms, and upset by hair loss scores. These items pertain to the side effects related to different treatment modalities, such as surgery, chemotherapy, or radiotherapy (15 items), dimensions of body image (4 items), sexuality (3 items), and future perspective (1 item). Each item is graded on a scale from 0 (not at all) to 4 (very much). According to the scores received, higher scores on the symptom-oriented scales correspond to higher levels of symptoms and higher scores on the functional scales represent higher levels of functioning. For easier interpretation, all scores from 1 to 4 or from 1 to 7 have been converted and range from 0 to 100, according to the EORTC Scoring manual [51].

Quality of Life was also assessed using the SF-36 questionnaire, which consists of 36 items divided into eight dimensions: physical functioning, role limitation due to physical health, bodily pain, general health perception, vitality, social functioning, and role limitation due to emotional health and mental health, which were interpreted separately, with higher scores on this scale indicating higher levels of health [29].

### Statistical analysis

The Shapiro-Wilk test was applied to analyze the sample distribution. Initially, in order to identify the similarity of both groups at baseline, the Student’s t-test for independent samples was used. The analysis assessed the intervention effects at 3, 6, and 9 months compared to the values at baseline, based on intention-to-treat principles [2].

In the longitudinal analysis, Linear General Mixed Models, two-way ANOVA was used to compare the CG and EX for quality of life. When a significant difference was observed, a Bonferroni post hoc test was conducted. For all measured variables, the estimated sphericity was verified according to the Mauchly’s W test and the Greenhouse-Geisser correction was used when necessary. Effect Size was considered low, moderate, and high and represented by a Cohen’s d greater than 0.2, 0.5, and 0.8, respectively [12]. The data were analyzed using SPSS (version 24) and Statsoft Statistic software (version 10).

## Results

The sociodemographic and clinical characteristics of participants are demonstrated in Table 1. No differences in baseline measures were found between the two groups. Retention in the study of women in the exercise group was 94% and in the control group 78%. Adherence to the combined training was 83%, being considered a very expressive and positive figure.

**Table 1:** Baseline sociodemographic and clinical characteristics of older breast cancer survivors

Variables	Exercise Group (*n* = 18)	Control Group (*n* = 18)	*p* value
Age	63.2 ± 7.1	66.6 ± 9.6	0.23
Total Mass (kg)	28.9 ± 5.2	31.5 ± 6.2	0.19
Height (cm)	154.1 ± 6.7	153.1 ± 4.5	0.33
BMI	66.9 ± 10.3	72.3 ± 13.1	0.18
Level of Schooling (%)			
No schooling	11.8	22.2	
Basic education	35.2	27.8	0.73
High school	23.6	27.8	
Higher education	29.4	22.2	
Marital Status (%)			
Single	11.1	5.6	
Married	61.1	55.6	0.53
Divorced	5.6	0	
Widowed	22.2	38.9	
Occupation (%)			
Housewife	55.6	50	
Works	5.6	16.7	0.27
Retired	38.9	33.3	
Children (%)			
No	5.6	5.6	0.82
yes	94.4	94.4	
Cancer Stage (%)			
I	50	58.8	
II	33.3	23.6	0.74
III	17.6	17.6	
Type of Surgery (%)			
Partial mastectomy	47.4	58.8	0.73
Total mastectomy	52.6	41.2	
General Treatment Received (%)			
Chemotherapy	66.6	69.3	0.31
Radiotherapy	77.8	75.2	0.10
Chemotherapy and Radiotherapy	50	38.9	0.50
Time of use of AI (months)	19.3 ± 8.3	17.9 ± 11.2	0.66
Number of diseases (%)			
2 diseases	27.8	16.7	0.53
More than 2 diseases	72.8	83.3	
Medicine used in a continuous manner			
2 medicine	22.2	24.8	0.97
More than 2 medicine	77.8	75.2	

*BMI* body mass index, *AI* aromatase inhibitors; numerical variables expressed as mean and standard deviation. Categorical variables expressed as %. No statistically significant baseline differences between groups

Regarding the effects of combined training on quality of life using the SF36 questionnaire, we observed a group x time interaction for the following variables: general health perception (*p* = 0.01), physical functioning (*p* = 0.008), physical health (*p* < 0.001), social functioning (*p* = 0.02), bodily pain (*p* < 0.001), and vitality (*p* < 0.001). A group x time interaction was not observed only for emotional health (*p* = 0.41) and mental health (*p* = 0.31). The post hoc analysis revealed that the exercise group significantly improved in comparison to the control group for almost all variables related to quality of life after 6 and 9 months of training, as shown in Table 2. Effect size was higher for almost all quality of life variables.

**Table 2:** Comparison between cancer survivors exercise group and cancer survivors control group in the quality of life variables of the SF36 questionnaire from baseline to nine months of combined training

Variables	Exercise Group (*n* = 18)			Control Group (*n* = 18)			Time *p*	Group *p*	Time x group interaction *p*	Effect size Cohen’d
	Baseline Mean ± SD	6 month Mean ± SD	9 months Mean ± SD	Baseline Mean ± SD	6 month Mean ± SD	9 months Mean ± SD				
General health perception	84.9 ± 10.8	84.9 ± 10.8	96.4 ± 4.7*^a,b^	83.8 ± 9.2	83.8 ± 9.2	87.3 ± 10.3	< 0.001	0.18	0.01	1.14
Physical health	75.8 ± 13.4	87.5 ± 12.7*^a^	93.9 ± 8.8*^a^	73.9 ± 11.5	70.8 ± 14.8	75.2 ± 12.6	< 0001	0.001	< 0.001	1.72
Physical functioning	77.8 ± 14.5	97.2 ± 8.08*^a^	98.6 ± 5.8*^a^	79.2 ± 12.8	84.7 ± 19.4	83.3 ± 14.8	< 0001	0.009	0.008	1.32
Emocional health	75.9 ± 27.6	81.6 ± 16.9	96.3 ± 10.7	68.6 ± 31.2	72.2 ± 23.5	77.9 ± 19.7	0.008	0.04	0.41	1.16
Social functioning	79.2 ± 14.9	95.8 ± 7.6^a^	96.7 ± 7.6^*a^	79.9 ± 11.1	87.1 ± 10.2	81.7 ± 17.7	< 0.001	0.005	0.02	1.10
Bodily pain	65.1 ± 12.6	86.9 ± 20.5*^a^	91.3 ± 11.8*^a^	61.1 ± 17.9	65.6 ± 22.1	56.1 ± 13.6	< 0.001	< 0.001	< 0.001	2.76
Vitality	80.6 ± 6.8	90.0 ± 10.4*^a^	92.8 ± 14.5*^a^	80.3 ± 6.5	78.6 ± 13.9	69.7 ± 15.8^a,b^	0.18	0.001	< 0.001	1.52
Mental health	84.6 ± 8.5	92.9 ± 9.5	85.6 ± 13.3	79.9 ± 8.6	82.2 ± 10.0	77.3 ± 8.4	0.006	0.002	0.31	0.75

Note: Post hoc analysis time x group interaction: * = statistically significant difference between groups and ^a^ = Bonferroni’s post hoc with *p* < 0.05 compared to baseline and ^b^ = Bonferroni’s post hoc with *p* < 0.05 compared to 6 months. Values expressed as mean and standard deviation

Table 3 presents the differences in functional scales from baseline to 9 months of combined training in both groups using the EORTC QLQ-C30. There was a significant time x group interaction (*p* = 0.01), effect of time (*p* < 0.001), and difference between groups (*p* = 0.02) for the role functioning. The post hoc analyses showed that the exercise group demonstrated a significant increase in quality of life for the variable role functioning from baseline to 3 months (*p* < 0.001), conversely, the control group demonstrated a significant increase only after 6 months (*p* = 0.02). In addition, the exercise group maintained higher values post 9 months of training compared to the control group. For social, physical, cognitive, and emotional functioning there was only a main effect of time in relation to baseline (Table 3).

**Table 3:** Comparison between cancer survivors exercise group and cancer survivors control group in the global health status and functional scales - EORTC QLQ C30 from baseline to nine months of combined training

Variables	Exercise Group (*N* = 18)				Control Group (*N* = 18)				Time *p*	Group *p*	Time x group interaction *p*	Effect size Cohen’d
	Baseline Mean ± SD	3 month Mean ± SD	6 months Mean ± SD	9 month Mean ± SD	Baseline Mean ± SD	3 month Mean ± SD	6 month Mean ± SD	9 month Mean ± SD				
Physical functioning	76.9 ± 10.3	92.9 ± 4.9	95.2 ± 6.9	94.9 ± 5.5	70.2 ± 15.2	75.1 ± 18.4	78.7 ± 16.1	82.2 ± 9.3	< 0.001	< 0.001	0.14	1.66
Role functioning	71.6 ± 25.5	96.1 ± 9.3*^a^	94.1 ± 13.1^a^	99.9 ± 24.6*^a^	66.6 ± 19.9	73.3 ± 19.7	85.6 ± 16.5^a^	72.2 ± 26.4	< 0.001	0.02	0.01	1.05
Emotional functioning	64.7 ± 20.1	84.8 ± 23.2	82.3 ± 19.7	85.3 ± 10.8	60.5 ± 27.7	67.2 ± 24.1	73.3 ± 25.2	71.1 ± 23.5	0.006	0.02	0.56	0.78
Cognitive functioning	70.6 ± 30.9	91.2 ± 13.3	83.3 ± 18.6	75.5 ± 20.5	70.0 ± 24.5	75.6 ± 22.5	84.4 ± 19.3	68.9 ± 25.1	0.008	0.29	0.33	0.29
Social functioning	88.2 ± 21.1	99.0 ± 4.1	99.8 ± 4.5	98.2 ± 8.1	82.2 ± 11.7	86.7 ± 21.0	87.8 ± 16.1	87.8 ± 9.8	0.01	0.004	0.59	1.14
Global health status	70.6 ± 14.1	84.4 ± 9.8	91.6 ± 10.4	86.5 ± 13.3	66.6 ± 15.2	68.1 ± 15.2	76.6 ± 14.8	73.8 ± 11.1	< 0.001	< 0.001	0.24	1.04

Note: Post hoc analysis time x group interaction: * = statistically significant difference between groups and ^a^ = Bonferroni’s post hoc with *p* < 0.05 compared to baseline. Values expressed as mean and standard deviation

The differences in global health status from baseline to 9 months of combined training in both groups, also using the EORTC QLQ-C30, are also shown in Table 3. The global health status presented a main effect of time (*p* < 0.001) and difference between groups (*p* < 0.001), but no time x group interaction (*p =* 0.24).

The effect size was high for all variables except cognitive function which was considered low, demonstrating that the combined training was effective using the EORTC QLQ-C30 questionnaire, promoting significant improvements in the quality of life of women who participated in the exercise program (Table 3).

The comparison of measurements of health-related quality of life with EORTC QLQ-C30 - Symptom scales/items from baseline to 9 months of combined training in both groups are presented in Table 4. The quality of life symptom presented significant group x time interactions for pain (*p =* 0.001), fatigue (*p* < 0.001), and sleep disturbance (*p* = 0.04). Post hoc analysis revealed that the exercise group demonstrated decreased pain and fatigue symptoms after 3 months, 6 months, and 9 months in relation to baseline (*p <* 0.001), but not the control group. For sleep disturbance, the exercise group presented a significant decrease after 3 months (*p* = 0.03), 6 months (*p <* 0.001), and 9 months (*p <* 0.001) compared to baseline. The control group demonstrated decreases only after 9 months (*p =* 0.01). The effect size for the combined training group was considered high for pain and fatigue.

**Table 4:** Comparison between cancer survivors exercise group and cancer survivors control group in the symptom scales/items - EORTC QLQ - BR23 from baseline to nine months of combined training

Variables	Exercise Group (*N* = 18)				Control Group (*N* = 18)				Time *p*	Group *p*	Time x group interaction *p*	Effect size Cohen’d
	Baseline Mean ± SD	3 month Mean ± SD	6 months Mean ± SD	9 month Mean ± SD	Baseline Mean ± SD	3 month Mean ± SD	6 month Mean ± SD	9 month Mean ± SD				
Pain	35.3 ± 25.6	8.8 ± 11.9*^a^	7.8 ± 11.9*^a^	4.9 ± 7.8*^a^	40.0 ± 28.7	34.4 ± 26.3	31.1 ± 16.5	32.8 ± 20.6	< 0.001	0.04	0.001	1.47
Fatigue	16.3 ± 19.3	4.6 ± 11.8*^a^	1.3 ± 5.4*^a^	0.6 ± 2.7*^a^	25.9 ± 22.8	20.0 ± 17.9	24.4 ± 18.9	22.9 ± 15.8	0.009	0.08	< 0.001	1.96
Nausea and vomiting	2.9 ± 8.8	0.9 ± 4.1	0.52 ± 1.25	2.9 ± 8.8	10.0 ± 24.2	11.1 ± 24.1	4.4 ± 9.8	3.7 ± 5.8	0.17	0.12	0.14	0.11
Dyspnea	3.9 ± 16.2	1.9 ± 8.1	7.8 ± 22.1	3.9 ± 11.1	19.9 ± 30.3	20.0 ± 30.3	8.8 ± 26.6	6.7 ± 18.7	0.40	0.12	0.09	0.18
Sleep Disturbance	41.2 ± 36.4	17.6 ± 33.1*^a^	7.8 ± 14.6*^a^	7.8 ± 18.7^a^	31.1 ± 38.7	35.5 ± 38.7	26.6 ± 36.1	17.8 ± 33.1^a^	0.01	0.16	0.04	0.37
Constipation	7.8 ± 25.1	6.1 ± 12.6	6.7 ± 25.8	11.8 ± 28.7	15.5 ± 35.3	17.8 ± 37.5	6.7 ± 25.8	6.7 ± 18.68	0.82	0.29	0.34	0.21
Diarrhea	1.9 ± 8.1	1.9 ± 8.1	5.7 ± 24.2	3.9 ± 11.1	11.1 ± 27.2	13.5 ± 15.1	12.3 ± 13.8	10.5 ± 14.1	0.40	0.16	0.79	0.52
Appetite loss	15.9 ± 24.2	12.2 ± 1.1	10.0 ± 3.5	13.9 ± 6.2	15.5 ± 24.6	13.3 ± 27.6	12.2 ± 8.6	14.4 ± 7.2	0.16	0.38	0.10	0.03
Financial difficulties	5.9 ± 13.1	3.9 ± 11.1	1.9 ± 8.1	1.8 ± 2.2	10.1 ± 13.1	6.7 ± 25.8	6.8 ± 1.6	4.5 ± 4.8	0.25	0.13	0.71	0.73

Note: Post hoc analysis time x group interaction: * = statistically significant difference between groups and ^a^ = Bonferroni’s post hoc with *p* < 0.05 compared to baseline. Values expressed as mean and standard deviation

Table 5 presents the comparison of measurements of psychological parameters - EORTC QLQ - BR23 from baseline to 9 months of combined training in both groups. Body image presented a significant time x group interaction (*p* = 0.01), main effect of time (*p* = 0.001), and difference between groups (*p* = 0.006). The post hoc test revealed that the exercise group demonstrated improved body image after 3 months of combined training compared to baseline (*p* < 0.001) and after 6 months in relation to 3 months of training. For upset due to hair loss, the exercise group demonstrated a significant improvement after 3 months and 6 months (*p* = 0.02). For the control group, there was a significant improvement after 3 months (*p =* 0.03), however, this increased again after 6 months (*p =* 0.01). The effect size for the group that participated in the combined training was considered high for body image.

**Table 5:** Comparison between cancer survivors exercise group and cancer survivors control group in the psychological parameters - EORTC QLQ - BR23 from baseline to nine months of combined training

Variables	Exercise Group (*N* = 18)				Control Group (*N* = 18)				Time *p*	Group *p*	Time x group interaction *p*	Size effect Cohen’d
	Baseline Mean ± SD	3 months Mean ± SD	6 months Mean ± SD	9 months Mean ± SD	Baseline Mean ± SD	3 months Mean ± SD	6 months Mean ± SD	9 months Mean ± SD				
Body image	77.4 ± 14.7	87.7 ± 18.9	98.1 ± 3.6*^a^	97.1 ± 7.2*^a^	73.9 ± 17.8	78.9 ± 15.4	85.6 ± 19.5	73.3 ± 24.8	0.001	0.006	0.01	1.30
Sexual functioning	89.2 ± 16.6	91.2 ± 22.1	87.2 ± 18.2	94.1 ± 14.4	85.6 ± 26.6	90.0 ± 18.7	86.7 ± 22.0	94.4 ± 14.9	0.31	0.96	0.77	0.02
Sexual enjoyment	88.2 ± 28.7	88.3 ± 29.0	78.4 ± 26.2	86.3 ± 26.5	93.3 ± 13.8	95.6 ± 17.2	95.6 ± 17.2	93.3 ± 18.7	0.66	0.41	0.16	0.31
Future Perspective	54.9 ± 38.9	47.1 ± 37.4	74.5 ± 27.7	78.4 ± 37.1	55.5 ± 44.8	57.8 ± 36.6	55.6 ± 34.9	46.6 ± 43.3	0.34	0.05	0.28	0.79
Systemic therapy side effects	28.3 ± 15.7	12.0 ± 14.9	7.8 ± 12.7	16.5 ± 14.4	34.7 ± 24.8	20.1 ± 22.5	18.7 ± 15.8	22.1 ± 13.1	< 0.001	0.86	0.08	0.41
Breast symptoms	11.3 ± 12.1	4.4 ± 7.8	10.8 ± 14.9	10.3 ± 12.7	14.9 ± 21.5	16.2 ± 19.2	10.1 ± 18.2	11.3 ± 13.3	0.72	0.25	031	0.08
Arm symptoms	20.3 ± 19.7	12.4 ± 18.4	16.9 ± 28.8	18.9 ± 15.1	27.8 ± 32.8	27.8 ± 32.8	18.2 ± 21.6	24.6 ± 21.7	0.58	0.53	0.23	0.30
Upset by hair loss	27.4 ± 39.5	9.8 ± 25.7^a^	5.9 ± 13.1*^a^	17.6 ± 33.6	38.1 ± 43.1	23.8 ± 42.2	30.9 ± 33.2	33.3 ± 34.4	0.18	0.80	0.03	0.46

Note: Post hoc analysis time x group interaction: * = statistically significant difference between groups and ^a^ = Bonferroni’s post hoc with *p* < 0.05 compared to baseline. Values expressed as mean and standard deviation

## Discussion

Combined resistance and aerobic training can improve physical, psychological, and social functioning outcomes, with potential benefits for quality of life and health in older breast cancer survivors treated with AI, also contributing to public health actions. Evidence suggests that physical exercise improves factors important to quality of life in breast cancer, mainly related to the treatment side effects and patient-reported outcomes, such as the aromatase inhibitor-induced musculoskeletal syndrome, a reduced risk of breast cancer recurrence, and improved breast cancer-specific and all-cause mortality [43]. Our results are similar to a recent meta-analysis that evaluated the effectiveness of exercise on overall quality of life and by domains among adult post-treatment cancer survivors. The authors suggested that exercise may improve quality of life in physical and social aspects as well as symptoms resulting from the treatment, and, after the exercise program was completed, may reduce physiological problems [37].

Our study population is very specific, postmenopausal breast cancer survivors, undergoing AI treatment, in early breast cancer stages I-III. There are few studies with similar characteristics. Thomas et al. [52] examined the effects of 12 months of aerobic and resistance exercise versus usual care on changes in body composition in postmenopausal breast cancer survivors taking AI and the study outcomes suggest that exercise interventions may help to mitigate the negative side effects of AI and improve health outcomes in breast cancer survivors. The study from Arem et al. [4] showed that exercise can improve AI-associated arthralgia in breast cancer survivors undergoing AI. The clinical characteristics of the two studies were similar to our study; women, with an average age of 62 years, diagnosed with early breast cancer stages I to III, and average time on AI therapy of 1.9 years.

The study of Shobeiri et al. [50] also supports our findings. The authors conducted a randomized clinical trial over 10 weeks based on the EORTC QLQ-C30 and QLQ-BR23 with the objective of evaluating the role of aerobic exercise in quality of life among women suffering from breast cancer. The authors reported that aerobic training was associated with substantial development in global health status (*p* < 0.001) and increased scores for functions and symptoms (p < 0.001) in the exercise group in comparison with the control group, according to the mean of the scores.

The large clinical trial by Saarto et al. [48] also assessed quality of life using the EORTC QLQ-C30 and QLQ-BR23 questionnaires, aiming to determine whether 12-months of physical exercise training improves the quality of life and physical fitness of breast cancer survivors after adjuvant treatments. In contrast to our findings, the authors reported no significant differences between the exercise and control groups regarding changes in quality of life during the intervention. The authors stated some reasons for this finding: the study was not blinded, and could have motivated the control participants, generally increasing physical activity in both groups; the insensitivity of questionnaires, which are designed to evaluate the quality of life in cancer patients, not cancer survivors, added to which the patients started the study with high scores on the quality of life scales. On the other hand, the authors accentuated the potential of their study, particularly the large sample size which is the largest prospective randomized trial with a physical exercise intervention for breast cancer survivors published to date.

According to the quality of life health reports based on the SF36 and EORTC QLQ- C30 and BR23 questionnaires, the combined exercise program provided additional evidence of a powerful mechanism to improve many functions and decrease symptoms in breast cancer survivors when compared to the control group. We found high effect sizes for almost all quality of life variables and these outcomes demonstrated positive clinical relevance of combined exercise programs for breast cancer survivors using aromatase inhibitors. Our study reported improvements in functioning and this finding is important for maintaining independence, daily living activities, healthy aging, and reducing the effects of treatment, as this effect may continue for a long time after treatment. These findings are supported by a recent meta-analysis that reported improvements in quality of life through exercise in clinical trials with breast cancer survivors [54]. In addition, in the present study, we observed higher adherence for the exercise group, which could have improved strength and aerobic capacity and may have contributed to the improved functional status of the older breast cancer exercise group, as has been shown in several studies [13, 15, 36].

Treatments for breast cancer, such as chemotherapy, radiation, and endocrine therapy, can induce impairments and injuries in the locomotor system, such as, pain, lymphedema, and a reduction in shoulder motion and muscle strength, consequently influencing the ability to perform daily living activities [31, 41, 45]. All the women in our study were undergoing treatment with AI and this therapy is considered the best adjuvant therapy for postmenopausal woman with hormone receptor-positive breast cancer, as this can reduce breast cancer recurrence and metastasis, and improve overall survival [28]. However, treatment with AI inhibits the aromatization of androgens and their conversion into estrogens in peripheral fat tissues and tumor cells, leading to a marked reduction in plasma estrogen by blocking the aromatase cytochrome P450 enzyme. In addition, the collateral effects are associated with increased bone turnover, which leads to loss of bone mineral density, increased fracture rate [6, 53], and arthralgia, characterized by musculoskeletal symptoms of joint pain and muscle stiffness [8, 40].

The findings from the present study demonstrated that older breast cancer survivors can adapt to an exercise program, demonstrated by the high adherence rates, also suggesting that selected functions and symptoms, such as bodily pain, can be improved with a combined exercise program. Denysschen et al. [16] compared a home-based exercise program that combined upper and lower body resistance exercises with self-selected aerobic exercises over a short period (8 weeks) in breast cancer patients treated with AI and observed improved lower joint pain, quality of life, and functional performance. The authors concluded that exercise had a positive effect on reducing joint pain, improving functional performance and quality of life, and reducing depressive symptoms in breast cancer patients treated with AI.

The present findings showed that fatigue and sleep disturbance in breast cancer survivors improved with combined training and should be recommended for daily practice in older breast cancer survivors to decrease the effects of treatment and co-morbidities. Fatigue interferes in physical, cognitive, and occupational functions, and this symptom is an important and common side effect associated with cancer treatment, chemotherapy, and radiation therapy; about 35% of cancer survivors present cancer-related fatigue 1 to 5 years after finishing treatment [14, 42].

The physiological effects of aerobic or combined modality training have been demonstrated to minimize fatigue and improve cardiorespiratory condition in older breast cancer patients [24]. Knobf et al. [32] examined the effects of a six-month exercise intervention on physical and psychological symptoms and quality of life in a group of breast cancer survivors, through a questionnaire, and found decreased fatigue, pain, and depression, and improved quality of live for physical, emotional, and social function. Rogers et al. [46] performed a randomized controlled trial and showed that moderate intensity aerobic walking and resistance training with elastic bands improved sleep duration and quality in postmenopausal breast cancer survivors.

Our intervention contributed to improved body image, social functioning, and upset due to hair loss, problems associated with depression, related to the cancer [23] and effects of the treatment for older breast cancer survivors, impacting highly on psychosocial health and quality of life [7, 44]. The training in the present study was conducted in a group, and the women had the same disease, treatment, and issues (physical and emotional) due to cancer. Thus, practicing exercise together in the same situation, may have helped to improve positive perception and satisfaction with body image and upset due to hair loss, such as looking better, increasing self-esteem, feeling better, improving relationships with other people, and motivation for exercise practice and life, as they were able to share experiences, fears, and challenges [9, 21, 49].

Despite the importance of this study, the limitations include the lack of performance tests for measuring physical function, small sample size, and self-reported assessments of questionnaires, which are generally inferior to objective measures.

## Conclusion

In summary, this study demonstrated the potential benefits of combined aerobic plus resistance training on quality of life in older breast cancer survivors who were undergoing aromatase inhibitor therapy. Furthermore, this kind of program could be an important strategy to improve health and minimize the effects of breast cancer treatment.

## Abbreviations

AI: Aromatase inhibitor therapy
BMI: Body mass index
CG: Breast cancer survivors control group
EORTC QLQ-BR23: European Organization for Research and Treatment of Cancer - breast cancer module
EORTC QLQ-C30: European Organization for Research and Treatment of Cancer
ER +: Estrogen receptor positive
EX: Breast cancer survivors combined exercise training group
FACT-B: Functional assessment of cancer therapy-breast scale
HR: Maximum heart rate
QoL: Quality of life
SF-36: Quality of life questionnaire
